# Difference in transducin-like enhancer of split 1 protein expression between basal cell adenomas and basal cell adenocarcinomas - an immunohistochemical study

**DOI:** 10.1186/s13000-018-0726-8

**Published:** 2018-07-27

**Authors:** Yuzo Oyama, Haruto Nishida, Takahiro Kusaba, Hiroko Kadowaki, Motoki Arakane, Junpei Wada, Shogo Urabe, Takashi Hirano, Kenji Kawano, Masashi Suzuki, Shigeo Yokoyama, Tsutomu Daa

**Affiliations:** 1 Department of Diagnostic Pathology, Faculty of Medicine, Oita University, 1-1, Idaigaoka, Hasama-machi, Yufu, 879-5593 Japan; 2 Oita Preferectual Hospital, Oita, Japan; 3 Department of Otolaryngology, Faculty of Medicine, Oita University, Yufu, Japan; 4 Department of Oral and Maxillofacial Surgery, Faculty of Medicine, Oita University, Yufu, Japan

**Keywords:** TLE1, Basal cell adenoma, Basal cell adenocarcinoma, β-catenin

## Abstract

**Background:**

Basal cell adenoma (BCA) and basal cell adenocarcinoma (BCAC) are benign and malignant, basaloid salivary gland neoplasms, respectively. These tumors show a dual-cell proliferation of inner luminal/ductal cells and outer abluminal/myoepithelial or basal cells. The only difference between them is defined as a malignant morphology such as invasion. Recently, the nuclear expression of β-catenin and a catenin beta-1 (*CTNNB1*) mutation were found in BCA. Transducin-like enhancer of split 1 (TLE1) belongs to the Groucho/TLE family, and it functions in the “off” state in the Wnt/β-catenin signaling pathway. We hypothesized that if the dysregulation of the Wnt/β-catenin signaling pathway could be attributed to the tumorigenesis of BCA/BCAC, there might be differences in TLE1 expression between BCA and BCAC.

**Method:**

The study included 35 BCA and 4 BCAC cases. We performed immunohistochemistry to detect TLE1 and β-catenin and investigated the catenin beta-1 (*CTNNB1*) mutational profile among BCA and BCAC cases.

**Results:**

In BCA, the expression of TLE1 was confined to luminal cells of glandular structures, in contrast to the expression of β-catenin in abluminal cells. The BCA cases harbored *CTNNB1* gene mutations (12/35). In BCAC, luminal cell staining of TLE1 was identical to BCA in non-invasive areas (4/4) but indistinct in invasive areas (3/4). The BCAC cases were β-catenin positive for abluminal cells in both areas. The BCAC cases had CTNNB1 mutation (2/4) and the laser-captured microdissection allowed the separate collection of infiltrative and non-infiltrative areas to detect the same mutation.

**Conclusions:**

Immunohistochemical analysis for TLE1 can identify BCA and BCAC by luminal cell staining difference, especially indistinct luminal cell expression for TLE1 in invasive areas of BCAC. Moreover, TLE1 can be luminal/ductal cell markers.

## Background

A basal cell adenoma (BCA) is a benign salivary gland basal cell neoplasm (BCN) that shows a dual-cell proliferation of inner luminal/ductal cells and outer abluminal/ myoepithelial or basal cells [1]. It has tubular, solid, nested, trabecular, and membranous growth with nuclear palisading [1]. Currently, catenin beta-1 (*CTNNB1*) gene mutations are exclusively found in BCA [2]. The nuclear expression of β-catenin is considered a characteristic feature of BCA because other salivary gland tumors rarely show nuclear staining [2]. In the presence of Wnt signal stimulation or an activating CTNNB1 gene mutation, β-catenin stabilizes and moves into the nucleus and interacts with T-cell factor (TCF)/lymphoid enhancer factor (LEF), which represents the “on” state of the Wnt/β-catenin signaling pathway and leads to an increase in the transcription of the target gene associated with the pathway [3].

A basal cell adenocarcinoma (BCAC) is a malignant salivary gland BCN that possesses dual-cell proliferation similar to BCA [4]. Many authors differentiate BCAC from BCA by their malignant morphological features, such as invasion into surrounding tissues and perineural and angiolymphatic invasion [4–8]. Although BCAC is recognized as the malignant counterpart of BCA, it is difficult to distinguish BCAC from BCA in surgical pathology practice. Several studies have attempted to identify characteristic immunohistochemical markers for the diagnosis of BCAC [5–8]. In other aspects, BCAC is generally recognized to have “low-grade malignancy” in terms of good overall survival [9]; however, high-grade cases of BCAC (9%), some frequent recurrent cases (16.7–50%), and some fatal cases (2–5.6%) presented by several authors indicate the necessity for differentiating between BCA and BCAC [5, 7, 9, 10]. These findings suggest that there is some heterogeneity in biological behavior among BCAC cases.

Transducin-like enhancer of split 1 (TLE1) is a member of the Groucho (ortholog of *Drosophila*)/TLE gene family located at chromosome 9q21.32 and acts as a transcriptional corepressor [11]. The association of TLE1 and tumors was first detected in synovial sarcomas through gene expression profiling, and TLE1 was found to be profoundly involved in the Wnt/β-catenin signaling pathway in synovial sarcomas [12, 13]. Furthermore, TLE1 expression was associated with better prognosis in gastric cancer and human epidermal growth-factor receptor 2 (HER2)-positive or triple-negative breast cancer, and the clinical significance of the expression profile of TLE1 in several carcinomas was recently reported [11, 14]. To the best of our knowledge, there are no relevant reports regarding TLE1 expression in salivary gland neoplasms.

During the absence of the Wnt signal and impeded migration of β-catenin to the nucleus, TLE interacts with TCF/LEF and histone deacetylase (HDAC) instead of β-catenin and functions to repress the transcription of Wnt target genes, representing the “off” state of the Wnt/β-catenin signaling pathway [15]. We hypothesized that if the dysregulation of the Wnt/β-catenin signaling pathway could be attributed to the tumorigenesis of BCA/BCAC, there might be differences in TLE1 expression between BCA and BCAC. This is the first report on TLE1 expression from the perspective of Wnt/β-catenin signaling. Therefore, we performed immunohistochemical analyses of TLE1 and β-catenin and conducted a mutational analysis of the *CTNNB1* gene in BCA and BCAC cases to identify an approach to differentiate BCA from BCAC.

## Methods

### Case selection

This study was approved by the institutional review board of Oita University Hospital and Oita Prefecture Hospital. Thirteen-nine cases were retrieved from the archives of Oita University Hospital and Oita Prefecture Hospital. We reviewed their hematoxylin-eosin (HE) stained specimens according to the World Health Organization classification of head and neck tumors [1, 4]. The study cases included 35 BCA and 4 BCAC cases. The clinicopathological findings of the patients are summarized in Table 1 (BCA) and Table 2 (BCAC). In BCA cases, the mean patient age was 65.4 years (range, 35–89 years), and the mean lesion size was 22.8 mm (range, 8–60 mm). There was no sex predilection (male:female = 16:19), and the lesions occurred mainly in the parotid gland (parotid gland, 33 cases; submandibular gland, 2 cases). Additionally, no recurrence was noted (follow-up period: 0.5 month to 13 years). In BCAC cases, the mean patient age was 55.3 years (range, 24–70 years), and the mean lesion size was 32.0 mm (range, 15–53 mm). There was a female predilection (male:female = 0:4), and the lesions occurred solely in the parotid gland (parotid gland, 4 cases). None of the cases showed recurrence (follow-up period: 1 month to 5 years).

**Table 1:** Clinicopathological information of the 35 basal cell adenoma cases

Case No.	Sex	Origin	Size (mm)	TLE1 (luminal cell)	β-catenin (abluminal cell)	Ki67	Mitosis (/10HPF)	CTNNB1 mutation	Follow-up (Months)
1	F	P	11	++	–	3.4%	1/10	–	6
2	M	P	20	++	++	1.1%	1/10	+	6
3	F	P	10	+	++	1%	1/10	–	2
4	M	P	20	++	+	1.3%	0/10	+	6
5	F	P	16	++	++	1.1%	0/10	+	25
6	M	P	30	++	–	1.9%	1/10	–	32
7	F	P	30	++	- (cytoplasm+)	3%	1/10	–	38
8	F	P	20	++	++	0.5%	0/10	–	1
9	M	P	10	++	++	0.4%	1/10	–	38
10	M	P	20	++	+	1.1%	1/10	+	12
11	F	P	20	+	–	1.7%	0/10	–	43
12	M	P	20	++	++	1.5%	0/10	–	1
13	F	P	8	+	+	1.1%	1/10	–	28
14	F	P	35	–	–	1.0%	0/10	–	76
15	F	P	26	+	++	1.3%	0/10	–	18
16	M	S	44	+	–	0.9%	3/10	–	18
17	F	P	10	++	++	1.0%	1/10	+	141
18	F	P	15	+	+	2.3%	1/10	–	100
19	F	P	20	+	++	1.0%	1/10	–	7
20	F	P	60	+	+	2.4%	0/10	+	12
21	F	P	25	+	++	1.5%	0/10	+	65
22	M	P	20	- (old sample)	–	1.0%	0/10	+	43
23	F	S	10	++	–	1.7%	0/10	–	4
24	F	P	20	++	++	1.7%	0/10	+	1
25	M	P	50	++	–	3.8%	0/10	–	1
26	M	P	30	+	++	1.6%	1/10	–	1
27	F	P	16	++	++	2.5%	1/10	–	36
28	M	P	31	++	++	2.7%	1/10	–	2
29	M	P	17	+	++	1.2%	0/10	–	0.5
30	M	P	20	++	++	2.2%	1/10	–	0.5
31	M	P	31	++	++	2.3%	1/10	–	0.5
32	M	P	20	++	+	0.8%	1/10	+	NA
33	F	P	25	++	+	1.4%	1/10	+	36
34	F	P	23	++	++	1.8%	1/10	–	156
35	M	P	15	+	+	1.0%	1/10	+	2

*P parotid gland*, *S* submandibular gland, *HPF* high power field, *NA* not available

**Table 2:** Clinicopathological information of 4 basal cell adenocarcinoma cases

Case No.	Sex	Origin	Size (mm)	TLE1 (nuclear)	β-catenin (nuclear)	Ki67	Mitosis (/10HPF)	CTNNB1 Mutation	Prognosis (months)
1	F	P	20	luminal cell ++, but indistinct at infiltrative areas	abluminal cell ++	9.0%	3/10	+	Alive (18)
2	F	P	40	luminal cell +, but indistinct at infiltrative areas	abluminal cell ++	4.7%	4/10	+	Alive (24)
3	F	P	15	luminal cell ++	abluminal cell +	5.3%	4/10	–	Alive (60)
4	F	P	53	luminal cell ++ indistinct at infiltrative areas	abluminal cell ++	7.6%	9/10	–	Alive (1)

*P* parotid gland, *HPF* high-power field

### Immunohistochemistry and analysis

For immunostaining, we used the standard streptavidin-biotin complex method (SAB-PO kit; Nichirei, Tokyo, Japan). After cutting sections (2 μm) from formalin-fixed paraffin embedded (FFPE) tissues were deparaffinized and rehydrated with xylene, alcohol, and tap water. Endogenous peroxidase was inactivated by 3% hydrogen peroxide. Next, heat epitope-induced retrieval was carried out according to primary antibody conditioning (121 °C, 15 min). After antigen retrieval, blocking was performed, and the slides were incubated with primary antibodies (Table 3). Next, the slides were immersed with biotin-labeled anti-mouse or anti-rabbit antibodies and streptavidin peroxidase, visualized with diaminobenzidine, and counterstained with hematoxylin. Each specimen was evaluated by 3 authors (YO, HN, and TD). For TLE1, staining of more than 30% of the tumor cells was considered positive, and the staining intensity was categorized as follows: strongly positive (2+) or positive (1+, intensity as strong as that for normal salivary acinic or intercalated ductal cells). Nuclear staining was defined as positive for TLE1, β-catenin, and Ki67 staining. The Ki67 labeling index (LI) was calculated according to the number of Ki67-positive cells per 1000 cells. Cytoplasm staining was considered positive for αSMA staining. We used EMA and αSMA to identify the dual-cell differentiation of luminal and abluminal cells, respectively.

**Table 3:** Primary antibodies and their conditioning in this study

Antibody	Clone	Source	Dilution	Conditioning
TLE1	EPR9386 (2)	Abcam, Cambridge, UK	1:100	pH 9.0
β-catenin	17C2	Novocastra, Newcastle, UK	1:100	pH 6.0 overnight
Ki67	MIB-1	DAKO, Santa Clara, USA	1:50	pH 6.0
αSMA	1A4	DAKO, Santa Clara, USA	1:100	
EMA	E29	Nichirei Bioscience, Tokyo, Japan	Diluted	

### *CTNNB1* gene mutation analysis

All tumor samples were analyzed for *CTNNB1* gene mutations. We used 1–2 sections of 10-μm FFPE-cut specimens. Genomic DNA was extracted using the QIAmp DNA FFPE Tissue Kit (Qiagen, Hilden, Germany) according to the manufacturer’s instructions. Exon 3 of the *CTNNB1* gene was amplified by polymerase chain reaction (PCR) using primer sets (Table 4) and the AmpliTaq Gold fast PCR master mix (Applied Biosystems, Foster, CA). The PCR reaction protocol was as follows: 10 min at 95 °C, 35 cycles of 3 s at 96 °C, 3 s at 60 °C, and 5 s at 68 °C, and 10 s at 72 °C. We used 5 μl of the PCR product as a template after treatment with 2 μl of ExoSAP-IT ™ (Affymetrix, Santa Clara, CA) for PCR product cleanup. This reaction protocol was as follows: 15 min at 37 °C and 15 min at 80 °C. The products were then sequenced using the BigDye™ Terminator v3.1 Cycle sequencing kit (Applied Biosystems). This protocol was as follows: 1 min at 96 °C, 25 cycles of 1 s at 96 °C and 5 s at 50 °C, and 2 min at 60 °C. Forward or reverse primers were used for the sequencing reaction. The products were reacted with components of the DyeEX 2.0 Spin kit (Qiagen) to remove unincorporated dye terminators. Finally, sequence analysis was performed using the ABI PRISM 310 Genetic Analyzer (Applied Biosystems).

**Table 4:** Primers sequence for CTNNB1 gene amplification used in this study

Primer	Sequence
exon 3 F1	5′-GAACCAGACAGAAAAGCGGCTG-3′
exon 3 R1	5′-ACTCATACAGGACTTGGGAGG-3′
exon 3 F2	5′-AAAGTAACATTTCCAATCTACTAATG-3′
exon 3 R2	5′-AAAATCCCTGTTCCCACTCA-3′

### Laser-capture microdissection

Laser-capture microdissection (LCM) was applied for BCAC cases 1, 3, and 4. LCM was not applied in BCAC case 2 because there were few FFPE blocks. We collected 4–5 10-μm sections from the non-infiltrative or infiltrative areas of the tumor separately in a 0.5-ml microtube using Leica LMD6000 (Leica Microsystems, Wetzlar, Germany). We then performed *CTNNB1* gene mutation analysis for each specimen, as mentioned above.

## Results

### TLE1 expression in normal salivary glands

**Fig. 1:**
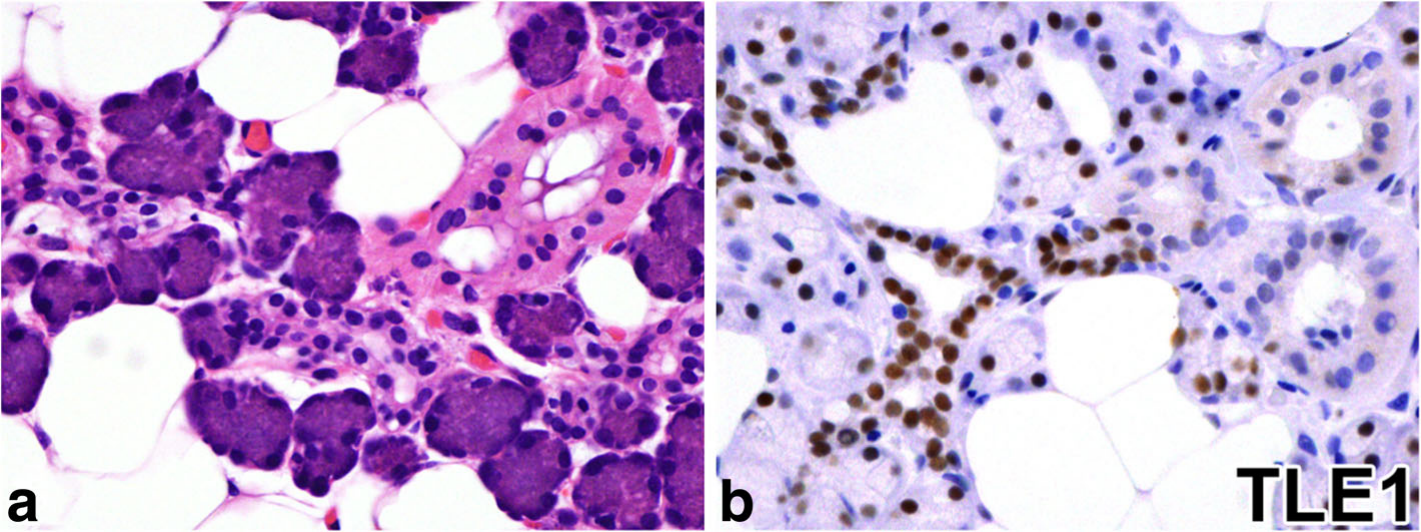
Normal salivary glands (**a**: hematoxylin–eosin staining, **b**: immunostaining for TLE1). Normal salivary gland forms ducto-acinar units composed of acini, intercalated ducts, striated ducts, and excretory ducts (**a**). Acinic cells and intercalated ductal cells are positive, and myoepithelial cells and excretory ductal cells are negative for TLE1 (**b**)

In normal salivary glands, acinic cells and intercalated ductal cells showed positive staining for TLE1 (Fig. 1).

### Histological findings of BCA and BCAC

**Fig. 2:**
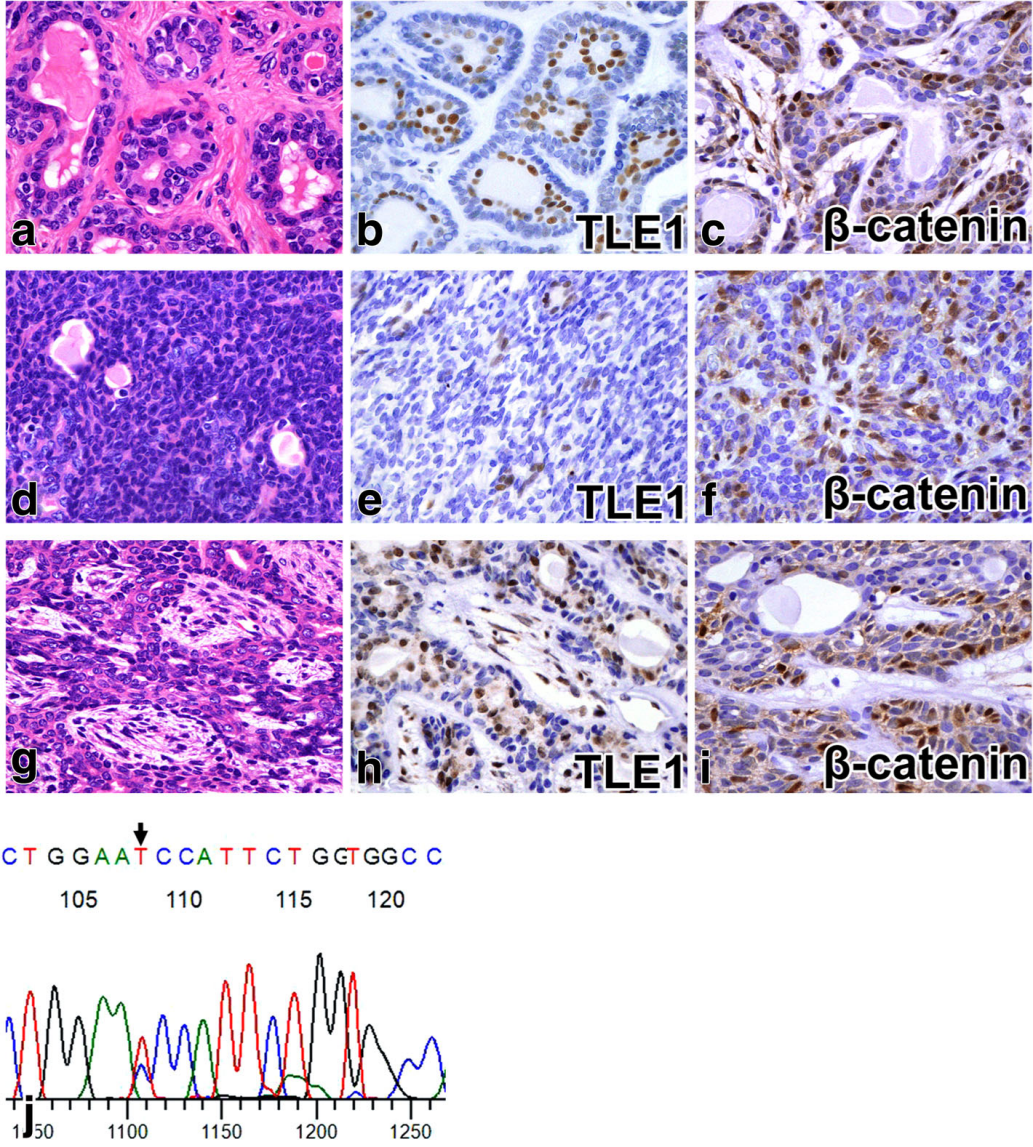
Basal cell adenoma (BCA). BCA of tubular (**a**-**c**), solid (**d**-**f**), trabecular (**g**-**i**) growth patterns with hematoxylin-eosin staining (**a**, **d**, and **g**), TLE1(**b**, **e**, and **h**), and β-catenin (**c**, **f**, and **i**) immunostaining, respectively, are shown. Luminal cells are positive for TLE1 (**b**, **e**, and **h**). Abluminal cells are positive for β-catenin (**c**, **f**, and **i**) in contrast to staining for TLE1. Mutational analysis for *CTNNB1* found an exon 3 mutation in 34% of basal cell adenomas (**j**, BCA case 10)

**Fig. 3:**
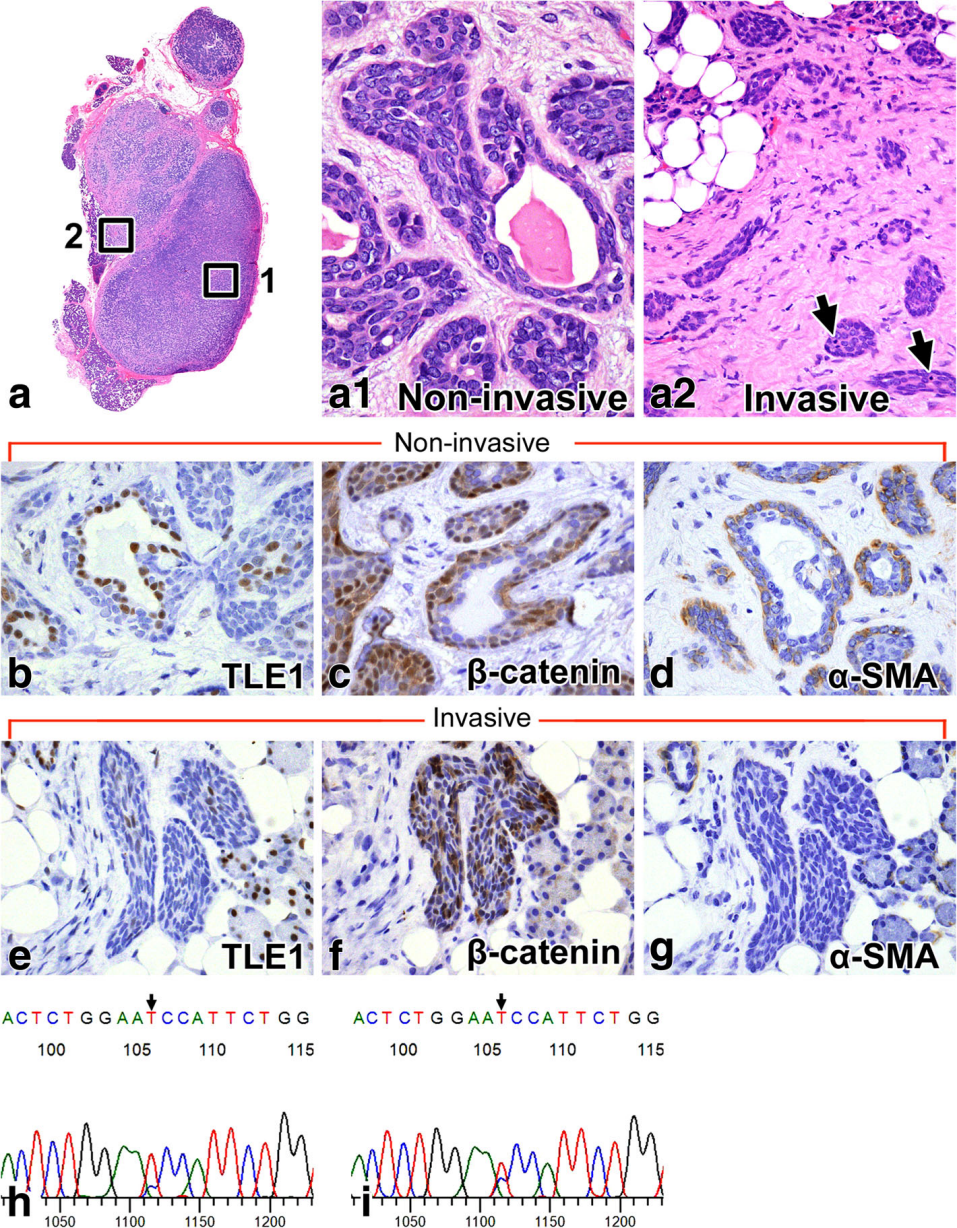
Basal cell adenocarcinoma (BCAC). BCAC histology (**a**: low magnification, a1: high magnification of square 1, a2: high magnification of square 2) with hematoxylin-eosin staining are shown. A multinodular tumor (**a**) forms a tubular, trabecular, solid growth pattern in non-invasive areas (a1), and small tumor nests infiltrate the surrounding parenchyma (a2). They show slightly increased mitotic activity (arrows in a2). BCAC of non-invasive areas (**b**-**d**) and invasive areas (**e**-**g**) with TLE1(**b**, **e**), β-catenin (**c**, **f**), and α-SMA (**d**, **g**) immunostaining, respectively, are shown. In non-invasive areas, luminal cells are positive for TLE1(**b**), abluminal cells are positive for β-catenin(**c**), and the lining of myoepithelial cells are positive for α-SMA(**d**). In invasive areas, luminal cells staining for TLE1(**e**) becomes indistinct, abluminal cells positivity for β-catenin(**f**) remains unchanged, and the α-SMA(**g**) lining myoepithelial cells disappear. Mutational analysis for *CTNNB1* detected the same mutation on both areas of the tumor (**h**: non-invasive areas, i: invasive areas, BCAC case 1)

BCA cases displayed various growth patterns, such as tubular, solid, and trabecular structures, and the tumor cells were basaloid cells with pale eosinophilic cytoplasm and oval nuclei and showed few mitoses (Fig. 2a, d and g). For the BCAC cases, we confirmed that they all showed malignant morphology, such as infiltration into the surrounding salivary gland or adipose tissue. BCAC cases showing solid, tubular, and small nested structures; the tumor cells were basaloid cells, were nearly identical to those in BCA cases and showed slightly increased mitotic counts in infiltrative lesions (mean: 5.0/10 high power fields [HPF]) (Fig. 3a, a1, and a2). None of the BCAC cases possessed the characteristic histological features of adenoid cystic carcinoma (ACC), including cribriform growth patterns or angular nucleus of tumor cells; thus, we excluded ACC.

### Expression of TLE1 and other proteins by immunostaining in BCA and BCAC

In BCA cases, the luminal cells of glandular structures were positive for TLE1 (33/35, 94%) (Fig. 2b, e and h), and the abluminal cells were positive for β-catenin (26/35, 74%) (Fig. 2c, f and i). In BCAC cases, TLE1 showed a luminal cell staining pattern (4/4, 100%) in non-infiltrative areas and indistinct or negative expression in infiltrative areas (Fig. 3b and e). In BCAC, the abluminal cells were positive for β-catenin in both areas (Fig. 3c and f), and the lining myoepithelial cells were positive for αSMA in non-infiltrative areas but disappeared in infiltrative areas (Fig. 3d and g). The Ki67 LI values were 1.6% (range, 0.4–3.8%), 6.7% (range, 4.7–9.0%) for BCA, and BCAC cases, respectively.

### *CTNNB1* gene mutation analysis

DNA was successfully extracted in 39 cases. Among the BCA cases, 12 of 35 cases (34%) had *CTNNB1* gene mutations (11 cases showed I35T mutation (Fig. 2j) and 1 case showed H36P mutation. Among the BCAC cases, 2 with I35T mutation were detected. The mutation could be found in both infiltrative and non-infiltrative areas (Fig. 3h and i).

## Discussion

Wnt/β-catenin signaling regulates postnatal development and regeneration of salivary gland and it is under in tight control because the dysregulation of it correlates with tumorigenesis [16, 17]. TLE1 functions in the “off” state in the Wnt/β-catenin signaling pathway [15]. In normal salivary glands, TLE1 showed staining for acinar and intercalated ductal cells, indicating that TLE1 expression was limited to “luminal” cells. β-catenin is generally expressed in the cytoplasm or cytomembrane in normal salivary grands [2], in contrast to the nuclear expression for TLE1, suggesting that salivary glands are normally in “off” state of Wnt/β-catenin signaling. BCA showed positive TLE1 expression in the luminal cells of glandular structures, regardless of the tumor growth patterns. This result is consistent with the theory that BCA originates from intercalated duct lesions [7], and this finding might suggest that tumor cells maintain an “off” state of the Wnt/β-catenin signaling pathway. The staining intensity of luminal cell staining of TLE1 in normal salivary gland and BCA cases was reduced with an increase in the age of the FFPE sample, possibly indicating that the staining was naïve expression. Interestingly, the TLE1 and β-catenin staining patterns were clearly separated (TLE1 for luminal cells and β-catenin for abluminal cells). Kawahara et al. comprehensively analyzed the β-catenin expression profile in salivary gland neoplasms, which showed a nuclear expression of basaloid myoepithelial cells that was exclusively found in BCA cases, and 52% of BCA cases had activating mutations of the *CTNNB1* gene [2]. Our results were consistent with the findings of previous reports regarding the nuclear expression of β-catenin and mutation of the *CTNNB1* gene identified in 74 and 34% of BCA cases, respectively; however, our mutation detection rate was slightly lower than their findings [2, 3, 8].

By contrast, the BCAC cases showed TLE1 staining for luminal cells, similar to that in BCA, in non-infiltrative areas but indistinct staining in infiltrative areas. BCAC is considered to arise mostly de novo or to progress from BCA in some cases [4, 5, 10]. TLE1 staining revealed antecedent BCA areas in all cases of BCAC. In this study, we could identify the BCAC, which some authors recognized as carcinoma ex monomorphic adenoma, carcinoma in basal cell adenoma, and BCAC evolution from a preexisting BCA [5, 10]. In BCAC, the obscure expression of TLE1 in infiltrative areas might suggest that these areas shifted the Wnt/β-catenin signaling pathway to the “on” state compared with non-infiltrative areas, resulting in malignancy characteristics such as invasion. Indistinct staining of αSMA lining myoepithelial cells in the invasive lesions supported this suggestion because the surrounding myoepithelial cells were considered tumor suppressors and were seen in both benign and in-situ salivary gland neoplasms but were rarely found in invasive neoplasms [18]. In BCAC, the nuclear expression of β-catenin in abluminal cells could be seen in both areas, and 50.0% (2/4) of the cases had *CTNNB1* gene mutations. The LCM allowed the separate collection of infiltrative and non-infiltrative areas, and this is the first report to document the same mutation of the *CTNNB1* gene in both areas. This finding strengthened the secondary theory and indicated that the adenoma-carcinoma sequence existed between BCA and BCAC.

Several features and immunohistochemical markers of preferentially suggestive BCAC have been proposed, although many of them remain ancillary observations [5–7]. BCAC displays various growth patterns (tubular, trabecular, solid), similar to BCA; however, many authors noted that solid patterns were frequently seen in BCAC cases [5–7, 10]. Nagao et al. found that BCAC cases showed increased mitotic figures (> 4/10 HPF) and a Ki67 LI (> 5%), and Wilson et al. suggested that mitotic rates above 3 per 10 HPF were more correlated with BCACs cases [5, 7]. Most of our BCAC cases showed solid growth patterns (3/4, 75%) and satisfied the condition of an increased mitotic count (> 3/10 HPF) and a Ki67 LI (> 5%). These findings are optional but helpful for identifying BCAC. Staining for TLE1 can be an additional approach for differentiating BCAC from BCA, especially indistinct luminal cell expression for TLE1 in invasive areas of BCAC.

Our study has limitations. The number of BCAC cases was small. Further studies are warranted to confirm the utility of TLE1 immunohistochemistry in salivary gland tumors.

## Conclusion

In conclusion, we successfully investigated the TLE1 and β-catenin expression profiles and the *CTNNB1* gene mutational status among BCA and BCAC cases. In BCA cases, TLE1 showed staining in luminal cells in contrast to β-catenin staining in abluminal cells. In BCAC, TLE1 showed luminal cell staining in non-infiltrative areas and indistinct staining in infiltrative areas. *CTNNB1* mutations were found in 34% of the BCA cases, 50% of the BCAC cases. We furthermore demonstrated the same mutation found in non-invasive and invasive areas in BCAC and indicated that the adenoma-carcinoma sequence exsisted between BCA and BCAC. Immunohistochemical analysis for TLE1 can help identify BCA and BCAC from its luminal cell staining difference, especially indistinct luminal cell expression for TLE1 in invasive areas of BCAC. Moreover, TLE1 can be luminal/ductal cell markers.

## Abbreviations

BCA: Basal cell adenoma
BCAC: Basal cell adenocarcinoma
BCN: Basal cell neoplasm
CTNNB1: Catenin-beta 1
FFPE: Formalin-fixed paraffin embedded
HDAC: Histone deacetylase
HER2: Human epidermal growth-factor receptor 2
HPF: High power fields
LCM: Laser-capture microdissection
LEF: Lymphoid enhancer factor
LI: Labeling index
TCF: T-cell factor
TLE1: Transducin-like enhancer of split 1
